# Crystal structure of the ternary silicide Gd_2_Re_3_Si_5_

**DOI:** 10.1107/S1600536814024234

**Published:** 2014-11-08

**Authors:** Vitaliia Fedyna, Roksolana Kozak, Roman Gladyshevskii

**Affiliations:** a Department of Inorganic Chemistry, Ivan Franko National University of Lviv, Kyryla i Mefodiya st. 6, UA-79005 Lviv, Ukraine; b Laboratory of Crystallography, Department of Materials, ETH Zurich, Vladimir-Prelog-Weg 5, CH-8093 Zurich, Switzerland

**Keywords:** crystal structure, gadolinium, rhenium, silicon, inter­metallic compound, ternary silicide

## Abstract

A single crystal of the title compound, the ternary silicide digadolinium trirhenium penta­silicide, Gd_2_Re_3_Si_5_, was isolated from an alloy of nominal composition Gd_20_Re_30_Si_50_ synthesized by arc melting and investigated by X-ray single-crystal diffraction. Its crystal structure belongs to the U_2_Mn_3_Si_5_ structure type. All atoms in the asymmetric lie on special positions. The Gd site has site symmetry *m*..; the two Mn atoms have site symmetries *m*.. and 2.22; the three Si atoms have site symmetries *m*.., ..2 and 4.. . The coordination polyhedra of the Gd atoms have 21 vertices, while those of the Re atoms are cubo­octa­hedra and 13-vertex polyhedra. The Si atoms are arranged as tricapped trigonal prisms, bicapped square anti­prisms, or 11-vertex polyhedra. The crystal structure of the title compound is also related to the structure types CaBe_2_Ge_2_ and W_5_Si_3_. It can be represented as a stacking of Gd-centred polyhedra of composition [GdSi_9_]. The Re atoms form infinite chains with an Re—Re distance of 2.78163 (5) Å and isolated squares with an Re—Re distance of 2.9683 (6) Å.

## Abstract 2

The crystal structure of this ternary silicide belongs to the U_2_Mn_3_Si_5_ structure type. The coordination polyhedra of the Gd atoms have 21 vertices, while those of the Re atoms are cubo­octa­hedra and 13-vertex polyhedra, and the Si atoms are arranged as tricapped trigonal prisms, bicapped square anti­prisms, or 11-vertex polyhedra.

## Chemical context   

Four structure types of composition *R*_2_ *T*_3_Si_5_ are known for the systems *R*–*T*–Si (*R* = rare-earth element, *T* = *d*-block element): U_2_Mn_3_Si_5_ (Yarmolyuk *et al.*, 1977 ▶) (Pearson symbol *tP*40, space group *P*4/*mnc*), U_2_Co_3_Si_5_ (Akselrud *et al.*, 1977 ▶) (*oI*40, *Ibam*), Nd_2_Os_3_Si_5_ (Rizzoli *et al.*, 2004 ▶) (*tP*48, *P*4/*mnc*) and Lu_2_Co_3_Si_5_ (Chabot & Parthé, 1985 ▶) (*mS*40, *C*2*/c*). The structure type U_2_Mn_3_Si_5_ has representatives in the systems *R*–Mn–Si (*R* = Y, Gd–Lu), *R*–Re–Si (*R* = Y, La–Nd, Sm, Gd–Tm), *R*–Fe–Si (*R* = Sc, Y, Sm, Gd–Lu), *R*–Ru–Si (*R* = Sm, Er, Lu), whereas the structure type U_2_Co_3_Si_5_ has been found in the systems *R*–Ru–Si (*R* = Tb, Er), *R*–Co–Si (*R* = Sc, Y, Ce, Gd–Er), *R*–Rh–Si (*R* = Y, La, Ce, Nd, Sm, Gd–Er), *R*–Ir–Si (*R* = Y, Ce, Tb, Lu), *R*–Ni–Si (*R* = Y, Ce, Nd, Sm, Gd–Tm), *R*–Pt–Si (*R* = Ce, Sm), and *R*–Pd–Si (*R* = Ce, Sm), the structure type Nd_2_Os_3_Si_5_ in the systems *R*–Os–Si (*R* = Nd, Eu), and the structure type Lu_2_Co_3_Si_5_ in the systems *R*–Co–Si (*R* = Y, Tb, Dy, Lu), *R*–Rh–Si (*R* = Y, Tb, Dy) and *R*–Ni–Si (*R* = Lu) (Villars & Cenzual, 2013 ▶).

## Structural commentary   

**Figure 1:**
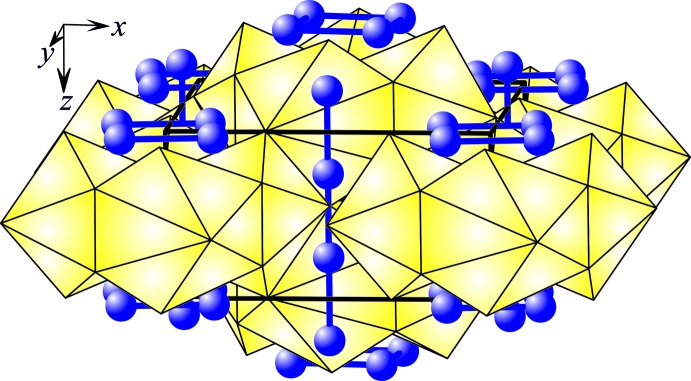
Stacking of Gd-centred polyhedra in the structure of the compound Gd_2_Re_3_Si_5_ with displacement ellipsoids drawn at the 99% probability level.

The existence of the compound Gd_2_Re_3_Si_5_ has been reported earlier (Bodak *et al.*, 1978 ▶). The unit-cell parameters were determined and the structure type was assigned. A complete investigation of the crystal structure by X-ray single crystal diffraction has now been undertaken. The coordination polyhedra of the Gd atoms have 21 vertexes, whereas those of the Re atoms are cubo­octa­hedra or 13-vertex polyhedra, and the Si atoms tricapped trigonal prisms, bicapped square anti­prisms, or 11-vertex polyhedra. The U_2_Mn_3_Si_5_-type structure is closely related to the structure type BaAl_4_ and its ordered derivative CaBe_2_Ge_2_. In particular, the U_2_Mn_3_Si_5_-type can be considered to be formed by one-dimensional structural fragments of the structure type CaBe_2_Ge_2_, running parallel to the direction [00l]. There also exists a relationship between the structure types U_2_Mn_3_Si_5_ and W_5_Si_3_. Fragments which can be viewed as deformed square anti­prisms are common to both structures. The crystal structure of Gd_2_Re_3_Si_5_ can also be represented as a stacking of Gd-centred polyhedra of composition [GdSi_9_], located at *z* = 0 and ½ (Fig. 1 ▶) (Parthé *et al.*, 1993 ▶). The Re atoms form infinite chains with an Re—Re distance of 2.78163 (5) Å and isolated squares with an Re—Re distance of 2.9683 (6) Å.

## Synthesis and crystallization   

An alloy of nominal atom percent composition Gd_20_Re_30_Si_50_ was synthesized from the high-purity elements by arc melting on a water-cooled copper plate under a purified argon atmos­phere, using titanium as a getter and a tungsten electrode. The weight loss during the sample preparation was less than 0.5% of the total mass (1 g). The alloy was placed into an Al_2_O_3_ crucible and inserted into a tantalum container, which was then sealed by welding, leaving the sample under an argon atmosphere. The sample, wrapped in tantalum foil, was heated to 1623 K in a muffle furnace at a rate of 200 K h^−1^, held at this temperature for 5 h and then cooled to room temperature at a rate of 50 K h^−1^.

## Refinement details   

A single crystal of well-defined shape was separated from the sample. The structure was solved by direct methods. The highest Fourier difference peak of 2.35 e Å^−3^ is at (0, 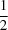, 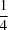), 0.00 Å away from atom Re2. The deepest hole (−2.44 e Å^−3^) is at (0.6045, 0.3985, 0), 1.52 Å away from the Gd atom. Details of the crystal parameters, data collection and the structure refinement details are summarized in Table 1 ▶.

## Supplementary Material

Crystal structure: contains datablock(s) I. DOI: 10.1107/S1600536814024234/fj2683sup1.cif

Structure factors: contains datablock(s) I. DOI: 10.1107/S1600536814024234/fj2683Isup2.hkl

CCDC reference: 1032373

Additional supporting information:  crystallographic information; 3D view; checkCIF report

## Figures and Tables

**Table 1:** Experimental details

Crystal data	
Chemical formula	Gd_2_Re_3_Si_5_
*M*_r_	1013.55
Crystal system, space group	Tetragonal, *P*4/*m* *n* *c*
Temperature (K)	293
*a*, *c* ()	10.95564(13), 5.56326(11)
*V* (^3^)	667.74(2)
*Z*	4
Radiation type	Mo *K*
(mm^1^)	74.55
Crystal size (mm)	0.16 0.10 0.02

Data collection	
Diffractometer	Agilent Xcalibur Onyx
Absorption correction	Analytical [*CrysAlis PRO* (Agilent, 2012 ▶; analytical numeric absorption correction using a multi-faceted crystal model (Clark Reid, 1995 ▶)]
*T*_min_, *T*_max_	0.015, 0.194
No. of measured, independent and observed [*I* > 2(*I*)] reflections	11378, 502, 481
*R*_int_	0.062
(sin /)_max_ (^1^)	0.692

Refinement	
*R*[*F* ^2^ > 2(*F* ^2^)], *wR*(*F* ^2^), *S*	0.025, 0.063, 1.17
No. of reflections	502
No. of parameters	31
_max_, _min_ (e ^3^)	2.35, 2.44

Computer programs: *CrysAlis PRO* (Agilent, 2012 ▶), *SHELXS97* and *SHELXL97* (Sheldrick, 2008 ▶), *WinGX* (Farrugia, 2012 ▶), *DIAMOND* (Brandenburg, 2006 ▶) and *publCIF* (Westrip, 2010 ▶).

## supplementary crystallographic information

### Crystal data

**Table:**

Gd_2_Re_3_Si_5_	*D*_x_ = 10.082 Mg m^−^^3^
*M**_r_* = 1013.55	Mo *K*α radiation, λ = 0.71073 Å
Tetragonal, *P*4/*m**n**c*	Cell parameters from 8564 reflections
Hall symbol: -P 4 2n	θ = 1.9–29.4°
*a* = 10.95564 (13) Å	µ = 74.55 mm^−^^1^
*c* = 5.56326 (11) Å	*T* = 293 K
*V* = 667.74 (2) Å^3^	Irregular, grey
*Z* = 4	0.16 × 0.10 × 0.02 mm
*F*(000) = 1692	

### Data collection

**Table:**

Agilent Xcalibur Onyx diffractometer	502 independent reflections
Radiation source: Enhance (Mo) X-ray Source	481 reflections with *I* > 2σ(*I*)
Graphite monochromator	*R*_int_ = 0.062
CCD scans	θ_max_ = 29.5°, θ_min_ = 2.6°
Absorption correction: analytical [*CrysAlis PRO* (Agilent, 2012; analytical numeric absorption correction using a multi-faceted crystal model (Clark & Reid, 1995)]	*h* = −14→14
*T*_min_ = 0.015, *T*_max_ = 0.194	*k* = −15→14
11378 measured reflections	*l* = −7→7

### Refinement

**Table:**

Refinement on *F*^2^	Primary atom site location: structure-invariant direct methods
Least-squares matrix: full	Secondary atom site location: difference Fourier map
*R*[*F*^2^ > 2σ(*F*^2^)] = 0.025	*w* = 1/[σ^2^(*F*_o_^2^) + (0.0342*P*)^2^ + 8.350*P*] where *P* = (*F*_o_^2^ + 2*F*_c_^2^)/3
*wR*(*F*^2^) = 0.063	(Δ/σ)_max_ < 0.001
*S* = 1.17	Δρ_max_ = 2.35 e Å^−^^3^
502 reflections	Δρ_min_ = −2.44 e Å^−^^3^
31 parameters	Extinction correction: *SHELXL97* (Sheldrick, 2008), Fc^*^=kFc[1+0.001xFc^2^λ^3^/sin(2θ)]^-1/4^
0 restraints	Extinction coefficient: 0.00016 (8)

### Special details

**Table:**

Experimental. Analytical numeric absorption correction using a multifaceted crystal model based on expressions derived by Clark & Reid (1995).
Geometry. All e.s.d.'s (except the e.s.d. in the dihedral angle between two l.s. planes) are estimated using the full covariance matrix. The cell e.s.d.'s are taken into account individually in the estimation of e.s.d.'s in distances, angles and torsion angles; correlations between e.s.d.'s in cell parameters are only used when they are defined by crystal symmetry. An approximate (isotropic) treatment of cell e.s.d.'s is used for estimating e.s.d.'s involving l.s. planes.
Refinement. Refinement of *F*^2^ against ALL reflections. The weighted *R*-factor *wR* and goodness of fit *S* are based on *F*^2^, conventional *R*-factors *R* are based on *F*, with *F* set to zero for negative *F*^2^. The threshold expression of *F*^2^ > σ(*F*^2^) is used only for calculating *R*-factors(gt) *etc*. and is not relevant to the choice of reflections for refinement. *R*-factors based on *F*^2^ are statistically about twice as large as those based on *F*, and *R*- factors based on ALL data will be even larger.

### Fractional atomic coordinates and isotropic or equivalent isotropic displacement parameters (Å^2^)

**Table:**

	*x*	*y*	*z*	*U*_iso_*/*U*_eq_	
Gd	0.26249 (5)	0.42271 (5)	0.0000	0.0187 (2)	
Re1	0.14676 (4)	0.12315 (4)	0.0000	0.01716 (18)	
Re2	0.0000	0.5000	0.2500	0.0173 (2)	
Si1	0.0267 (3)	0.3149 (3)	0.0000	0.0192 (6)	
Si2	0.17183 (18)	0.67183 (18)	0.2500	0.0145 (6)	
Si3	0.0000	0.0000	0.2567 (9)	0.0197 (9)	

### Atomic displacement parameters (Å^2^)

**Table:**

	*U*^11^	*U*^22^	*U*^33^	*U*^12^	*U*^13^	*U*^23^
Gd	0.0183 (3)	0.0199 (3)	0.0179 (3)	0.0004 (2)	0.000	0.000
Re1	0.0166 (3)	0.0172 (3)	0.0177 (3)	−0.00060 (16)	0.000	0.000
Re2	0.0169 (2)	0.0169 (2)	0.0180 (3)	0.0003 (2)	0.000	0.000
Si1	0.0207 (15)	0.0184 (15)	0.0184 (14)	0.0029 (12)	0.000	0.000
Si2	0.0130 (8)	0.0130 (8)	0.0174 (13)	0.0005 (10)	−0.0025 (8)	0.0025 (8)
Si3	0.0190 (13)	0.0190 (13)	0.021 (2)	0.000	0.000	0.000

### Geometric parameters (Å, º)

**Table:**

Gd—Si1	2.841 (4)	Re2—Re2^xiv^	2.7816 (1)
Gd—Si1^i^	2.9604 (12)	Re2—Re2^viii^	2.7816 (1)
Gd—Si1^ii^	2.9604 (12)	Re2—Gd^viii^	3.3047 (5)
Gd—Si3^iii^	3.053 (2)	Re2—Gd^xii^	3.3047 (5)
Gd—Si3^iv^	3.053 (2)	Re2—Gd^ii^	3.3047 (5)
Gd—Re1^ii^	3.1442 (3)	Si1—Re1^xi^	2.467 (4)
Gd—Re1^i^	3.1442 (3)	Si1—Re2^viii^	2.476 (3)
Gd—Si2^v^	3.163 (2)	Si1—Si2^viii^	2.585 (3)
Gd—Si2^vi^	3.163 (2)	Si1—Si2^xiii^	2.585 (3)
Gd—Si2^vii^	3.2202 (11)	Si1—Gd^i^	2.9604 (12)
Gd—Si2	3.2202 (11)	Si1—Gd^ii^	2.9604 (12)
Gd—Re2^viii^	3.3047 (5)	Si2—Re1^iii^	2.4837 (12)
Re1—Si1^ix^	2.467 (4)	Si2—Re1^xv^	2.4837 (12)
Re1—Si1	2.479 (3)	Si2—Si1^viii^	2.585 (3)
Re1—Si2^vi^	2.4838 (12)	Si2—Si1^ii^	2.585 (3)
Re1—Si2^v^	2.4838 (12)	Si2—Si2^xvi^	2.7816 (1)
Re1—Si3	2.539 (3)	Si2—Si2^vii^	2.7816 (1)
Re1—Si3^x^	2.539 (3)	Si2—Gd^iii^	3.163 (2)
Re1—Re1^xi^	2.9683 (6)	Si2—Gd^xv^	3.163 (2)
Re1—Re1^ix^	2.9684 (6)	Si2—Gd^xii^	3.2202 (11)
Re1—Gd^ii^	3.1442 (3)	Si3—Re1^x^	2.539 (3)
Re1—Gd^i^	3.1442 (3)	Si3—Re1^ix^	2.539 (3)
Re2—Si1^ii^	2.476 (3)	Si3—Re1^xi^	2.539 (3)
Re2—Si1^xii^	2.476 (3)	Si3—Si3^xvii^	2.707 (9)
Re2—Si1^viii^	2.476 (3)	Si3—Gd^ii^	3.053 (2)
Re2—Si1	2.476 (3)	Si3—Gd^xviii^	3.053 (2)
Re2—Si2^xiii^	2.662 (3)	Si3—Gd^xix^	3.053 (2)
Re2—Si2	2.662 (3)	Si3—Gd^xx^	3.053 (2)
			
Si1—Gd—Si1^i^	79.48 (8)	Si1^ii^—Re2—Gd^xii^	94.36 (7)
Si1—Gd—Si1^ii^	79.48 (8)	Si1^xii^—Re2—Gd^xii^	56.71 (8)
Si1^i^—Gd—Si1^ii^	139.97 (12)	Si1^viii^—Re2—Gd^xii^	59.57 (4)
Si1—Gd—Si3^iii^	152.94 (8)	Si1—Re2—Gd^xii^	168.30 (6)
Si1^i^—Gd—Si3^iii^	127.50 (10)	Si2^xiii^—Re2—Gd^xii^	115.730 (10)
Si1^ii^—Gd—Si3^iii^	77.03 (10)	Si2—Re2—Gd^xii^	64.270 (10)
Si1—Gd—Si3^iv^	152.94 (8)	Re2^xiv^—Re2—Gd^xii^	65.111 (4)
Si1^i^—Gd—Si3^iv^	77.03 (10)	Re2^viii^—Re2—Gd^xii^	114.889 (4)
Si1^ii^—Gd—Si3^iv^	127.50 (10)	Gd^viii^—Re2—Gd^xii^	74.405 (16)
Si3^iii^—Gd—Si3^iv^	52.64 (16)	Si1^ii^—Re2—Gd^ii^	56.71 (8)
Si1—Gd—Re1^ii^	105.17 (3)	Si1^xii^—Re2—Gd^ii^	94.36 (7)
Si1^i^—Gd—Re1^ii^	172.19 (7)	Si1^viii^—Re2—Gd^ii^	168.29 (6)
Si1^ii^—Gd—Re1^ii^	47.80 (6)	Si1—Re2—Gd^ii^	59.57 (4)
Si3^iii^—Gd—Re1^ii^	48.34 (7)	Si2^xiii^—Re2—Gd^ii^	64.270 (10)
Si3^iv^—Gd—Re1^ii^	96.88 (8)	Si2—Re2—Gd^ii^	115.730 (10)
Si1—Gd—Re1^i^	105.17 (3)	Re2^xiv^—Re2—Gd^ii^	65.111 (4)
Si1^i^—Gd—Re1^i^	47.80 (6)	Re2^viii^—Re2—Gd^ii^	114.889 (4)
Si1^ii^—Gd—Re1^i^	172.19 (7)	Gd^viii^—Re2—Gd^ii^	128.540 (19)
Si3^iii^—Gd—Re1^i^	96.88 (8)	Gd^xii^—Re2—Gd^ii^	130.223 (8)
Si3^iv^—Gd—Re1^i^	48.34 (7)	Si1^ii^—Re2—Gd	59.57 (4)
Re1^ii^—Gd—Re1^i^	124.42 (2)	Si1^xii^—Re2—Gd	168.30 (6)
Si1—Gd—Si2^v^	81.12 (7)	Si1^viii^—Re2—Gd	94.36 (7)
Si1^i^—Gd—Si2^v^	79.33 (7)	Si1—Re2—Gd	56.71 (8)
Si1^ii^—Gd—Si2^v^	129.87 (7)	Si2^xiii^—Re2—Gd	115.731 (10)
Si3^iii^—Gd—Si2^v^	104.01 (5)	Si2—Re2—Gd	64.269 (10)
Si3^iv^—Gd—Si2^v^	81.50 (4)	Re2^xiv^—Re2—Gd	114.888 (4)
Re1^ii^—Gd—Si2^v^	95.05 (3)	Re2^viii^—Re2—Gd	65.112 (4)
Re1^i^—Gd—Si2^v^	46.377 (11)	Gd^viii^—Re2—Gd	130.223 (8)
Si1—Gd—Si2^vi^	81.12 (7)	Gd^xii^—Re2—Gd	128.540 (19)
Si1^i^—Gd—Si2^vi^	129.87 (7)	Gd^ii^—Re2—Gd	74.406 (16)
Si1^ii^—Gd—Si2^vi^	79.33 (7)	Re1^xi^—Si1—Re2	122.21 (11)
Si3^iii^—Gd—Si2^vi^	81.50 (4)	Re1^xi^—Si1—Re2^viii^	122.21 (11)
Si3^iv^—Gd—Si2^vi^	104.01 (5)	Re2—Si1—Re2^viii^	68.34 (9)
Re1^ii^—Gd—Si2^vi^	46.377 (11)	Re1^xi^—Si1—Re1	73.76 (10)
Re1^i^—Gd—Si2^vi^	95.05 (3)	Re2—Si1—Re1	139.17 (9)
Si2^v^—Gd—Si2^vi^	52.16 (4)	Re2^viii^—Si1—Re1	139.17 (9)
Si1—Gd—Si2^vii^	94.12 (8)	Re1^xi^—Si1—Si2^viii^	58.84 (9)
Si1^i^—Gd—Si2^vii^	49.23 (7)	Re2—Si1—Si2^viii^	99.03 (11)
Si1^ii^—Gd—Si2^vii^	99.13 (6)	Re2^viii^—Si1—Si2^viii^	63.42 (8)
Si3^iii^—Gd—Si2^vii^	102.67 (6)	Re1—Si1—Si2^viii^	119.61 (12)
Si3^iv^—Gd—Si2^vii^	80.58 (5)	Re1^xi^—Si1—Si2^xiii^	58.84 (9)
Re1^ii^—Gd—Si2^vii^	135.25 (3)	Re2—Si1—Si2^xiii^	63.42 (8)
Re1^i^—Gd—Si2^vii^	86.902 (11)	Re2^viii^—Si1—Si2^xiii^	99.03 (11)
Si2^v^—Gd—Si2^vii^	128.074 (8)	Re1—Si1—Si2^xiii^	119.61 (12)
Si2^vi^—Gd—Si2^vii^	175.18 (3)	Si2^viii^—Si1—Si2^xiii^	65.09 (9)
Si1—Gd—Si2	94.12 (8)	Re1^xi^—Si1—Gd	156.27 (14)
Si1^i^—Gd—Si2	99.13 (6)	Re2—Si1—Gd	76.51 (9)
Si1^ii^—Gd—Si2	49.23 (7)	Re2^viii^—Si1—Gd	76.51 (9)
Si3^iii^—Gd—Si2	80.58 (5)	Re1—Si1—Gd	82.50 (10)
Si3^iv^—Gd—Si2	102.67 (6)	Si2^viii^—Si1—Gd	137.86 (11)
Re1^ii^—Gd—Si2	86.902 (11)	Si2^xiii^—Si1—Gd	137.86 (11)
Re1^i^—Gd—Si2	135.25 (3)	Re1^xi^—Si1—Gd^i^	84.88 (7)
Si2^v^—Gd—Si2	175.18 (3)	Re2—Si1—Gd^i^	141.66 (11)
Si2^vi^—Gd—Si2	128.075 (8)	Re2^viii^—Si1—Gd^i^	74.27 (2)
Si2^vii^—Gd—Si2	51.18 (2)	Re1—Si1—Gd^i^	69.99 (6)
Si1—Gd—Re2^viii^	46.77 (6)	Si2^viii^—Si1—Gd^i^	70.63 (3)
Si1^i^—Gd—Re2^viii^	46.16 (6)	Si2^xiii^—Si1—Gd^i^	132.78 (12)
Si1^ii^—Gd—Re2^viii^	95.63 (6)	Gd—Si1—Gd^i^	87.06 (7)
Si3^iii^—Gd—Re2^viii^	149.01 (2)	Re1^xi^—Si1—Gd^ii^	84.88 (7)
Si3^iv^—Gd—Re2^viii^	118.95 (7)	Re2—Si1—Gd^ii^	74.27 (2)
Re1^ii^—Gd—Re2^viii^	141.358 (16)	Re2^viii^—Si1—Gd^ii^	141.66 (11)
Re1^i^—Gd—Re2^viii^	92.088 (9)	Re1—Si1—Gd^ii^	69.99 (6)
Si2^v^—Gd—Re2^viii^	103.62 (3)	Si2^viii^—Si1—Gd^ii^	132.78 (12)
Si2^vi^—Gd—Re2^viii^	127.27 (3)	Si2^xiii^—Si1—Gd^ii^	70.63 (3)
Si2^vii^—Gd—Re2^viii^	48.14 (5)	Gd—Si1—Gd^ii^	87.06 (7)
Si2—Gd—Re2^viii^	72.31 (3)	Gd^i^—Si1—Gd^ii^	139.97 (12)
Si1^ix^—Re1—Si1	163.76 (10)	Re1^iii^—Si2—Re1^xv^	131.08 (12)
Si1^ix^—Re1—Si2^vi^	62.95 (8)	Re1^iii^—Si2—Si1^viii^	170.53 (14)
Si1—Re1—Si2^vi^	104.06 (9)	Re1^xv^—Si2—Si1^viii^	58.21 (7)
Si1^ix^—Re1—Si2^v^	62.95 (8)	Re1^iii^—Si2—Si1^ii^	58.21 (7)
Si1—Re1—Si2^v^	104.06 (9)	Re1^xv^—Si2—Si1^ii^	170.53 (14)
Si2^vi^—Re1—Si2^v^	68.11 (4)	Si1^viii^—Si2—Si1^ii^	112.58 (18)
Si1^ix^—Re1—Si3	96.85 (6)	Re1^iii^—Si2—Re2	114.46 (6)
Si1—Re1—Si3	96.56 (7)	Re1^xv^—Si2—Re2	114.46 (6)
Si2^vi^—Re1—Si3	107.81 (8)	Si1^viii^—Si2—Re2	56.29 (9)
Si2^v^—Re1—Si3	159.37 (6)	Si1^ii^—Si2—Re2	56.29 (9)
Si1^ix^—Re1—Si3^x^	96.85 (6)	Re1^iii^—Si2—Si2^xvi^	55.947 (19)
Si1—Re1—Si3^x^	96.56 (7)	Re1^xv^—Si2—Si2^xvi^	124.054 (19)
Si2^vi^—Re1—Si3^x^	159.37 (6)	Si1^viii^—Si2—Si2^xvi^	122.55 (5)
Si2^v^—Re1—Si3^x^	107.81 (8)	Si1^ii^—Si2—Si2^xvi^	57.45 (5)
Si3—Re1—Si3^x^	68.46 (18)	Re2—Si2—Si2^xvi^	90.0
Si1^ix^—Re1—Re1^xi^	143.30 (8)	Re1^iii^—Si2—Si2^vii^	124.054 (19)
Si1—Re1—Re1^xi^	52.94 (8)	Re1^xv^—Si2—Si2^vii^	55.947 (19)
Si2^vi^—Re1—Re1^xi^	141.12 (5)	Si1^viii^—Si2—Si2^vii^	57.45 (5)
Si2^v^—Re1—Re1^xi^	141.12 (5)	Si1^ii^—Si2—Si2^vii^	122.55 (5)
Si3—Re1—Re1^xi^	54.22 (4)	Re2—Si2—Si2^vii^	90.0
Si3^x^—Re1—Re1^xi^	54.22 (4)	Si2^xvi^—Si2—Si2^vii^	180.0
Si1^ix^—Re1—Re1^ix^	53.30 (8)	Re1^iii^—Si2—Gd^iii^	76.01 (6)
Si1—Re1—Re1^ix^	142.94 (8)	Re1^xv^—Si2—Gd^iii^	66.41 (5)
Si2^vi^—Re1—Re1^ix^	106.47 (5)	Si1^viii^—Si2—Gd^iii^	112.27 (8)
Si2^v^—Re1—Re1^ix^	106.47 (5)	Si1^ii^—Si2—Gd^iii^	118.78 (6)
Si3—Re1—Re1^ix^	54.22 (4)	Re2—Si2—Gd^iii^	140.83 (3)
Si3^x^—Re1—Re1^ix^	54.22 (4)	Si2^xvi^—Si2—Gd^iii^	63.919 (19)
Re1^xi^—Re1—Re1^ix^	90.0	Si2^vii^—Si2—Gd^iii^	116.083 (19)
Si1^ix^—Re1—Gd^ii^	116.473 (18)	Re1^iii^—Si2—Gd^xv^	66.41 (5)
Si1—Re1—Gd^ii^	62.214 (12)	Re1^xv^—Si2—Gd^xv^	76.01 (6)
Si2^vi^—Re1—Gd^ii^	67.22 (4)	Si1^viii^—Si2—Gd^xv^	118.78 (6)
Si2^v^—Re1—Gd^ii^	127.12 (2)	Si1^ii^—Si2—Gd^xv^	112.27 (8)
Si3—Re1—Gd^ii^	63.95 (8)	Re2—Si2—Gd^xv^	140.83 (3)
Si3^x^—Re1—Gd^ii^	123.79 (8)	Si2^xvi^—Si2—Gd^xv^	116.083 (19)
Re1^xi^—Re1—Gd^ii^	73.988 (16)	Si2^vii^—Si2—Gd^xv^	63.919 (19)
Re1^ix^—Re1—Gd^ii^	112.077 (11)	Gd^iii^—Si2—Gd^xv^	78.35 (7)
Si1^ix^—Re1—Gd^i^	116.473 (18)	Re1^iii^—Si2—Gd	79.226 (13)
Si1—Re1—Gd^i^	62.214 (12)	Re1^xv^—Si2—Gd	120.171 (7)
Si2^vi^—Re1—Gd^i^	127.12 (2)	Si1^viii^—Si2—Gd	94.29 (9)
Si2^v^—Re1—Gd^i^	67.22 (4)	Si1^ii^—Si2—Gd	60.14 (5)
Si3—Re1—Gd^i^	123.79 (8)	Re2—Si2—Gd	67.59 (5)
Si3^x^—Re1—Gd^i^	63.95 (8)	Si2^xvi^—Si2—Gd	115.588 (10)
Re1^xi^—Re1—Gd^i^	73.988 (16)	Si2^vii^—Si2—Gd	64.411 (10)
Re1^ix^—Re1—Gd^i^	112.077 (11)	Gd^iii^—Si2—Gd	148.88 (7)
Gd^ii^—Re1—Gd^i^	124.42 (2)	Gd^xv^—Si2—Gd	74.63 (2)
Si1^ix^—Re1—Gd	110.57 (8)	Re1^iii^—Si2—Gd^xii^	120.171 (7)
Si1—Re1—Gd	53.19 (8)	Re1^xv^—Si2—Gd^xii^	79.226 (13)
Si2^vi^—Re1—Gd	60.75 (5)	Si1^viii^—Si2—Gd^xii^	60.14 (5)
Si2^v^—Re1—Gd	60.75 (5)	Si1^ii^—Si2—Gd^xii^	94.29 (9)
Si3—Re1—Gd	136.39 (6)	Re2—Si2—Gd^xii^	67.59 (5)
Si3^x^—Re1—Gd	136.39 (6)	Si2^xvi^—Si2—Gd^xii^	64.411 (10)
Re1^xi^—Re1—Gd	106.13 (2)	Si2^vii^—Si2—Gd^xii^	115.588 (10)
Re1^ix^—Re1—Gd	163.87 (2)	Gd^iii^—Si2—Gd^xii^	74.63 (2)
Gd^ii^—Re1—Gd	73.474 (15)	Gd^xv^—Si2—Gd^xii^	148.88 (7)
Gd^i^—Re1—Gd	73.474 (15)	Gd—Si2—Gd^xii^	135.19 (9)
Si1^ii^—Re2—Si1^xii^	111.66 (9)	Re1—Si3—Re1^x^	111.54 (18)
Si1^ii^—Re2—Si1^viii^	120.57 (15)	Re1—Si3—Re1^ix^	71.55 (9)
Si1^xii^—Re2—Si1^viii^	97.03 (13)	Re1^x^—Si3—Re1^ix^	71.55 (9)
Si1^ii^—Re2—Si1	97.03 (13)	Re1—Si3—Re1^xi^	71.55 (9)
Si1^xii^—Re2—Si1	120.57 (15)	Re1^x^—Si3—Re1^xi^	71.55 (9)
Si1^viii^—Re2—Si1	111.66 (9)	Re1^ix^—Si3—Re1^xi^	111.54 (18)
Si1^ii^—Re2—Si2^xiii^	119.72 (8)	Re1—Si3—Si3^xvii^	124.23 (9)
Si1^xii^—Re2—Si2^xiii^	60.28 (8)	Re1^x^—Si3—Si3^xvii^	124.23 (9)
Si1^viii^—Re2—Si2^xiii^	119.72 (8)	Re1^ix^—Si3—Si3^xvii^	124.23 (9)
Si1—Re2—Si2^xiii^	60.28 (8)	Re1^xi^—Si3—Si3^xvii^	124.23 (9)
Si1^ii^—Re2—Si2	60.28 (8)	Re1—Si3—Gd^ii^	67.712 (11)
Si1^xii^—Re2—Si2	119.72 (8)	Re1^x^—Si3—Gd^ii^	151.41 (5)
Si1^viii^—Re2—Si2	60.28 (8)	Re1^ix^—Si3—Gd^ii^	129.93 (3)
Si1—Re2—Si2	119.72 (8)	Re1^xi^—Si3—Gd^ii^	81.779 (12)
Si2^xiii^—Re2—Si2	180.0	Si3^xvii^—Si3—Gd^ii^	63.68 (8)
Si1^ii^—Re2—Re2^xiv^	55.83 (4)	Re1—Si3—Gd^xviii^	81.779 (12)
Si1^xii^—Re2—Re2^xiv^	55.83 (4)	Re1^x^—Si3—Gd^xviii^	129.93 (3)
Si1^viii^—Re2—Re2^xiv^	124.17 (4)	Re1^ix^—Si3—Gd^xviii^	67.712 (11)
Si1—Re2—Re2^xiv^	124.17 (4)	Re1^xi^—Si3—Gd^xviii^	151.41 (5)
Si2^xiii^—Re2—Re2^xiv^	90.0	Si3^xvii^—Si3—Gd^xviii^	63.68 (8)
Si2—Re2—Re2^xiv^	90.0	Gd^ii^—Si3—Gd^xviii^	78.66 (6)
Si1^ii^—Re2—Re2^viii^	124.17 (4)	Re1—Si3—Gd^xix^	151.41 (5)
Si1^xii^—Re2—Re2^viii^	124.17 (4)	Re1^x^—Si3—Gd^xix^	67.712 (11)
Si1^viii^—Re2—Re2^viii^	55.83 (4)	Re1^ix^—Si3—Gd^xix^	81.779 (12)
Si1—Re2—Re2^viii^	55.83 (4)	Re1^xi^—Si3—Gd^xix^	129.93 (3)
Si2^xiii^—Re2—Re2^viii^	90.0	Si3^xvii^—Si3—Gd^xix^	63.68 (8)
Si2—Re2—Re2^viii^	90.0	Gd^ii^—Si3—Gd^xix^	127.36 (16)
Re2^xiv^—Re2—Re2^viii^	180.0	Gd^xviii^—Si3—Gd^xix^	78.66 (6)
Si1^ii^—Re2—Gd^viii^	168.29 (6)	Re1—Si3—Gd^xx^	129.93 (3)
Si1^xii^—Re2—Gd^viii^	59.57 (4)	Re1^x^—Si3—Gd^xx^	81.779 (12)
Si1^viii^—Re2—Gd^viii^	56.71 (8)	Re1^ix^—Si3—Gd^xx^	151.41 (5)
Si1—Re2—Gd^viii^	94.36 (7)	Re1^xi^—Si3—Gd^xx^	67.712 (11)
Si2^xiii^—Re2—Gd^viii^	64.270 (10)	Si3^xvii^—Si3—Gd^xx^	63.68 (8)
Si2—Re2—Gd^viii^	115.730 (10)	Gd^ii^—Si3—Gd^xx^	78.66 (6)
Re2^xiv^—Re2—Gd^viii^	114.889 (4)	Gd^xviii^—Si3—Gd^xx^	127.36 (16)
Re2^viii^—Re2—Gd^viii^	65.111 (4)	Gd^xix^—Si3—Gd^xx^	78.66 (6)

Symmetry codes: (i) −*y*+1/2, −*x*+1/2, *z*−1/2; (ii) −*y*+1/2, −*x*+1/2, *z*+1/2; (iii) −*x*+1/2, *y*+1/2, −*z*+1/2; (iv) *x*+1/2, −*y*+1/2, *z*−1/2; (v) −*y*+1, *x*, −*z*; (vi) −*y*+1, *x*, *z*; (vii) *x*, *y*, −*z*; (viii) −*x*, −*y*+1, −*z*; (ix) *y*, −*x*, −*z*; (x) −*x*, −*y*, −*z*; (xi) −*y*, *x*, *z*; (xii) *y*−1/2, *x*+1/2, −*z*+1/2; (xiii) −*x*, −*y*+1, *z*; (xiv) −*x*, −*y*+1, −*z*+1; (xv) *y*, −*x*+1, −*z*; (xvi) *x*, *y*, −*z*+1; (xvii) −*x*, −*y*, −*z*+1; (xviii) −*x*+1/2, *y*−1/2, −*z*+1/2; (xix) *y*−1/2, *x*−1/2, −*z*+1/2; (xx) *x*−1/2, −*y*+1/2, *z*+1/2.
